# Transdiagnostic effect of contamination disgust on asceticism

**DOI:** 10.1192/j.eurpsy.2021.1637

**Published:** 2021-08-13

**Authors:** G. Santarelli, M. Innocenti, V. Gironi, V. Faggi, F. Galassi, G. Castellini, V. Ricca

**Affiliations:** 1 Human Health Sciences, University of Florence, Firenze, Italy; 2 Human Health Sciences, University of Florence, firenze, Italy

**Keywords:** Asceticism, anxiety disorders, eating disorders, disgust

## Abstract

**Introduction:**

There is a growing literature suggesting disgust plays a major role in religiosity. Asceticism is a personality trait characterized by abstinence from sensual pleasures, often for the purpose of pursuing spiritual goals. Since few studies suggested that higher disgust levels may lead to greater fear of sin, contamination disgust may serve as effective mechanisms for inflated scrupulosity.

**Objectives:**

We aimed to investigate the role of contamination disgust on a specific religious belief in two clinical groups: eating disorders and anxiety disorders.

**Methods:**

We enrolled 84 patients admitted in the Psychiatric Unit of Careggi with diagnosis of Anxiety Disorders (AD) and Eating Disorders (ED). We administered them: Disgust Scale Revised (DS-R) and Eating Disorders Inventory 2 (EDI-2)

**Results:**

A t-test for independent samples between AD patients and ED patients showed no difference in DS-R contamination disgust subscale (t=1.437, p=0.153), while significantly higher EDI-2 asceticism scores were detected in ED patients (t=2.452, p=0.010). An ANCOVA model having EDI2 Asceticism subscale as dependent variable, and DS contamination disgust subscale, diagnosis, and interaction between contamination disgust and diagnosis was estimated. The overall model was significant (F(1,136)=4.854, p=0.003) and accounted for 9.7% of variance of EDI2 Asceticism subscale (R^2^=0.097). The effect of contamination disgust was positive and significant (β=0.302, t=2.781, p=0.006), and accounted for 5.4% of variance of EDI2 Asceticism (partial η^2^=0.054). No effect was detected for diagnosis (F(1,136)=0.012, p=0.912) or interaction between contamination disgust and diagnosis (F(1,136)=1.346, p=0.248).

**Figure:**
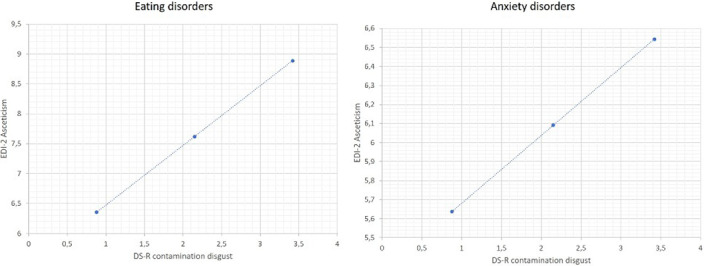

**Conclusions:**

Contamination disgust may have a trans-diagnostic effect on asceticism and may be a possible driver for specific religious behaviors.

**Disclosure:**

No significant relationships.

